# Abdominal Aortic Endothelial Dysfunction Occurs in Female Mice With Dextran Sodium Sulfate-Induced Chronic Colitis Independently of Reactive Oxygen Species Formation

**DOI:** 10.3389/fcvm.2022.871335

**Published:** 2022-04-07

**Authors:** Hao Wu, Tingzi Hu, Linfang Zhang, Xiujuan Xia, Xuanyou Liu, Qiang Zhu, Meifang Wang, Zhe Sun, Hong Hao, Yuqi Cui, Alan R. Parrish, De-Pei Li, Michael A. Hill, Canxia Xu, Zhenguo Liu

**Affiliations:** ^1^Center for Precision Medicine and Division of Cardiovascular Medicine, University of Missouri School of Medicine, Columbia, MO, United States; ^2^Department of Gastroenterology, Third Xiangya Hospital, Central South University, Changsha, China; ^3^Dalton Cardiovascular Research Center, University of Missouri, Columbia, MO, United States; ^4^Department of Medical Pharmacology and Physiology, University of Missouri School of Medicine, Columbia, MO, United States

**Keywords:** endothelial dysfunction, colitis, ROS, inflammatory bowel disease, aorta

## Abstract

**Background and Objective:**

Inflammatory bowel disease (IBD) produces significant local and systemic inflammation with increased reactive oxygen species (ROS) formation. IBD Patients are at an increased risk for developing endothelial dysfunction and cardiovascular diseases. The present study tested the hypothesis that IBD impairs aortic endothelial function via ROS formation and investigate potential sex-related differences.

**Methods and Results:**

Acute and chronic colitis models were induced in male and female C57BL/6 mice with dextran sodium sulfate (DSS) treatment. Aortic wall stiffness, endothelial function, and ROS levels, as well as serum levels of pro-inflammatory cytokines were evaluated. Acetylcholine (Ach)-induced endothelium-dependent relaxation of abdominal aorta without perivascular adipose tissue (PVAT) was significantly reduced in female mice, not males, with chronic colitis without a change in nitroglycerin-induced endothelium-independent relaxation. PVAT effectively preserved Ach-induced relaxation in abdominal aorta of female mice with chronic colitis. Aortic peak velocity, maximal intraluminal diameters, pulse wave velocity, distensibility and radial strain were preserved in mice with both acute and chronic colitis. Although pro-inflammatory cytokines levels were increased in mice with acute and chronic colitis, aortic ROS levels were not increased.

**Conclusion:**

The data demonstrate that abdominal aortic endothelial function was attenuated selectively in female mice with chronic colitis independent of ROS formation. Further, PVAT played an important role in preserving endothelial function in female mice with chronic colitis.

## Introduction

Inflammatory bowel disease (IBD), including Crohn’s disease (CD) and ulcerative colitis (UC), is characterized by chronic and recurrent intestinal inflammation and is associated with significant extra-intestinal manifestations (EIMs) including arthropathy and arthritis, metabolic bone disease, skin disease, hepato-pancreato-biliary disease, renal disease, and cardiovascular disease (CVD) (1, 2). Several studies have suggested that patients with IBD are at an increased risk for developing CVD especially in young women (3, 4), however, the underlying mechanisms are poorly defined.

Endothelial dysfunction is closely associated with the development and progression of CVD including atherosclerosis and hypertension (5). Studies have shown that endothelium-dependent flow-mediated vasodilation is significantly decreased in patients with IBD (6). Microvascular endothelial dysfunction as measured using pulse arterial tonometry (PAT) has been reported in IBD patients with decreased PAT indices (7), and increased aortic stiffness (an indicator for vascular endothelial dysfunction and an independent predictor of cardiovascular events) (8). Reactive oxygen species (ROS) contribute to significant endothelial dysfunction and subsequent development and progression of CVD, especially atherosclerosis (9). IBD is a chronic and recurrent condition with significant local (intestine) and systemic inflammation, and significant increases in pro-inflammatory cytokines including tumor necrosis factor (TNF)-α, interleukin (IL)-1β, and IL-6 (10). While this altered cytokine profile could be expected to increase ROS production, leading to endothelial dysfunction (11, 12) it is however, unclear if aortic endothelial function is impaired in IBD, and if there are sex-dependent differences.

Perivascular adipose tissue (PVAT) could release a variety of chemokines (including CCL2, CCL5, and CX3CL1) and adipocytokines that may have a significant impact on endothelial function via macrophage-mediated mechanism or anti-inflammatory activities (13, 14). It is known that aorta is heterogenous in its developmental origin, gene expression, and functionality (15). The present study was designed to test the hypothesis that experimental IBD leads to aortic endothelial cell dysfunction (thoracic and abdominal aorta) and excessive arterial stiffness through a mechanism related to increased ROS formation. There were four objectives: (1) to determine if IBD could decrease aortic endothelial function (as assessed by acetylcholine-mediated relaxation) both *in vivo* and *ex vivo*; (2) to determine if a sex difference was present in IBD-induced endothelial dysfunction; (3) to define the contribution of ROS formation to IBD-induced endothelial dysfunction; and (4) to investigate the role of PVAT in IBD-induced aortic endothelial dysfunction. As IBD likely exhibits temporal relationships, both acute (7 days) and chronic (51 days) mouse models were used to achieve the objectives, using dextran sodium sulfate (DSS)-induced colitis in both male and female mice for the study.

## Materials and Methods

### Animals

All animal experiments were conducted in accordance with the “Guide for the Care and Use of Laboratory Animals of US National Institutes of Health”. The experimental protocols were reviewed and approved by the Institutional Animal Care and Use Committee of the University of Missouri, Columbia, MO, United States (protocol number 9227). Six-week-old C57BL/6 male and female mice were purchased from Jackson Laboratory (Bar Harbor, ME, United States), and were housed under standard laboratory conditions (22 ± 1°C, 12:12-h light/dark cycle).

### Acute and Chronic DSS-Induced Colitis Mouse Models

Colitis was induced by oral administration of 2.5% DSS (MP Biomedicals, Santa Ana, CA, United States). DSS solution was made fresh every other day. Mice for the acute model were randomly assigned into four groups with 5–8 mice in each group, and for the chronic model 8–10 mice were included in each group: (1) female control, (2) female with DSS treatment, (3) male control, and (4) male with DSS treatment. For acute DSS-induced colitis, mice were treated for 7 days with DSS, and were euthanized on day 8 of DSS treatment. For chronic DSS-induced colitis, mice were treated with three cycles of DSS (7 days of treatment with 14 days of drinking water between each cycle) and subsequently euthanized on day 51 as described (16).

### Assessment of Colitis Severity

All mice were weighed daily and assessed for stool consistency and fecal occult blood (Germaine Laboratories, San Antonio, TX, United States). The disease activity index (DAI) was obtained using a scoring system for each mouse as described (17). At the termination of each experiment colon length was measured from the ileocecal junction to the anal verge. Colon tissues were fixed in 10% formalin for 24 h, rehydrated in graded alcohol, hyalinized with xylene, embedded in paraffin, and cut into 5 μm thick tissue sections of. The colon tissue preparations were stained with hematoxylin and eosin (H&E), and examined using an upright microscope (Leica DM5500B, Wetzlar, Germany). The level of inflammation in colon tissue was determined using a scoring system that included degrees of tissue damage and lamina propria inflammatory cell infiltration as described (16).

### Analyses of Plasma Cytokines

After fully isoflurane anesthesia, retroorbital blood was collected in K3EDTA micro tubes (Sarstedt, Nümbrecht, Germany) and plasma separated by centrifugation (3,000 *g* for 10 min at 4°C). Plasma samples were kept at -80°C until analysis. The levels of plasma cytokines were determined by multiplex immunoassay with a BioPlex 200 Mouse Cytokine Array/Chemokine Array (Eve Technologies, Calgary, Canada) (18).

### Blood Pressure Measurements

Blood pressure was measured non-invasively on conscious mice using a CODA volume pressure recording tail-cuff system (Kent Scientific Corporation, Torrington, CT, United States). Systolic, diastolic, and mean blood pressures were recorded.

### TransAbdominal Ultrasound Imaging of Abdominal Aorta

Transabdominal ultrasound imaging of the abdominal aorta was conducted for each mouse at baseline and at days 8, 22, 36, and 50 after DSS treatment under general anesthesia with oxygen (1 L/min) and vaporized isoflurane (1.5% vol/vol). Adequate levels of anesthesia were confirmed by the absence of withdrawal reflexes via toe pinching. The animals were placed on a heated imaging stage in the supine position. Body temperature, heart rate, and respiratory rate were continuously monitored during imaging. Abdominal hair was removed by applying hair removal cream followed by cleaning with wet gauze prior to abdominal aorta measurements. Warm ultrasound gel was applied to the abdominal wall for placement of ultrasound probe (MS400, 18–38 MHz) to collect B-mode, M-mode, PW doppler mode, as well as ECG based Kilohertz Visualization (EKV) mode images, using a high-resolution ultrasound imaging system (Vevo 2100, FUJIFILM VisualSonics Inc., Bothell, WA, United States). Peak blood flow velocity within abdominal aorta was quantified using PW doppler mode. Maximal aortic intraluminal diameters (MILD) were measured using M mode. Pulse wave velocity (PWV), distensibility, and radial strain were measured in the abdominal aorta with EKV mode using VevoVasc software as described (19).

### Aortic Endothelial Function Studies

Thoracic and abdominal aorta were carefully excised, dissected, and placed in ice-cold physiological saline solution (PSS) containing (in mM): 130 NaCl, 4.7 KCl, 1.18 KH_2_PO_4_, 1.17 MgSO_4_, 14.9 NaHCO3, 5.5 Glucose, 0.026 EDTA, and 1.6 CaCl_2_, pH 7.4, gassed with 95% O_2_ and 5% CO_2_. Thoracic and abdominal aorta (with and without PVAT) were carefully cut into rings (2 mm in length) under a stereo-dissection microscope (20). The aortic rings were mounted to a multi-wire myograph system (620M; Danish Myo Technology, Aarhus, Denmark). The organ chambers were filled with 5 ml PSS at 37°C and aerated continuously with carbogen (95% O_2_ + 5% CO_2_). Based on vessel length/tension relationships, a preload tension of 10 and 12.5 mN/mm was applied to thoracic and abdominal aortic rings, respectively. The rings were allowed to equilibrate for 1 h with replacement of PSS every 20 min. To determine vascular contractility, aortic rings were incubated with high potassium PSS containing (in mM): 74.7 NaCl, 60 KCl, 1.18 KH_2_PO_4_, 1.17 MgSO_4_, 14.9 NaHCO3, 5.5 Glucose, 0.026 EDTA, and 1.6 CaCl_2_ (pH 7.4). After adequate washout with PSS, a dose-response curve to endothelium-dependent vasodilator acetylcholine (Ach, 10^–9^ to 10^–5^ M, accumulative) and a dose-response curve to endothelium-independent vasodilator nitroglycerin (NTG, 10^–9^ to 10^–5^ M) for each aortic ring after submaximal precontraction with phenylephrine (PE, 10^–6^ M).

### Measurement of ROS Formation Using Dihydroethidium Assay

Thoracic and abdominal aorta were cleaned of perivascular tissue (especially PVAT) and vertically embedded in Tissue-Tek optimal cutting temperature (OCT) compound (Sakura Finetek, Torrance, CA, United States), and frozen in liquid nitrogen immediately. Liver tissue was embedded in OCT compound and frozen in liquid nitrogen with subsequent assessment of whether ROS levels were increased in the liver of mice with chronic DSS-induced colitis. Frozen cross-sections (5 μm in thickness) of aorta and liver were prepared and incubated with 5 μM dihydroethidium (DHE; Molecular Probes, Eugene, OR, United States) for 15 min as described (21). Images were obtained using a fluorescence microscope (Olympus CKX53, Tokyo, Japan), and analyzed with Image J (NIH, Bethesda, MD, United States) software.

### Statistical Analysis

All data were presented as mean ± SEM, and analyzed using an unpaired, two-tailed Student’s *t*-test or two-way ANOVA followed by Bonferroni correction. All statistical analyses were conducted using Prism 8.0 software (GraphPad Software Inc., La Jolla, CA, United States). A two-sided *p* < 0.05 was considered statistically significant.

## Results

### Assessment of Acute and Chronic DSS-Induced Colitis Models

**FIGURE 1:**
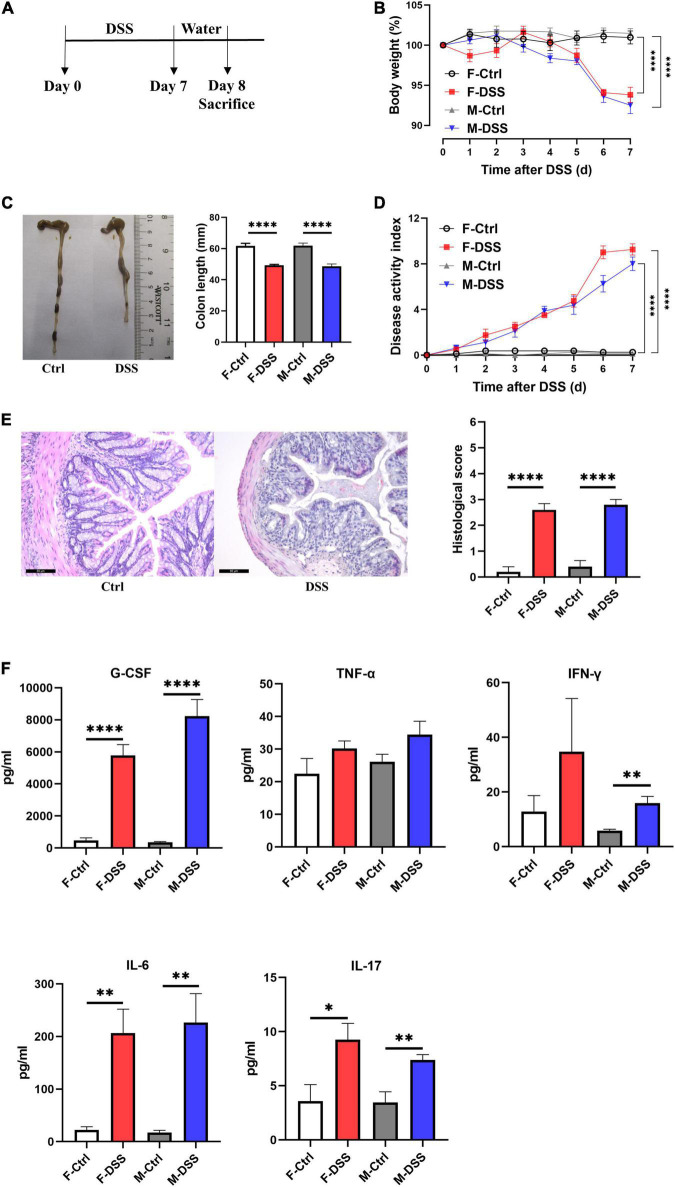
Both female and male mice with acute DSS-induced colitis displayed significant inflammation in the colon and systemic inflammation. **(A)** Experimental scheme illustrating DSS treatment protocol for acute models. Changes in **(B)** body weight and **(D)** disease activity index (DAI) during DSS treatment. **(C)** Colon length and representative photographs for colon tissue in DSS-treated and control mice. **(E)** Representative images of H&E staining of colon tissue (×200; scale bar, 50μm), and summary of histological score. **(F)** Levels of plasma cytokines in male and female mice with acute colitis model. Data are expressed as mean ± SEM. *p < 0.05, **p < 0.01, ****p < 0.0001, in unpaired 2-tailed Student’s *t*-test **(B–F)**, *n* = 5–8 mice each group. F and M, female and male mice, respectively. G-CSF, granulocyte colony-stimulating factor; TNF-α, tumor necrosis factor alpha; IFN-γ, interferon gamma; IL, interleukin.

All mice (both male and female) treated with 2.5% DSS for 7 days developed acute colitis (Figure 1A), as indicated by significant decreases in body weight (Figure 1B), colon length (Figure 1C), and significant increases in disease activity index (DAI) score (reflecting the severity of colitis) compared to control mice (Figure 1D). Increased colonic inflammation was also confirmed by histological analysis, showing mucosal erosions and lamina propria inflammatory cell infiltration in colon tissues of mice with acute colitis, compared to non-DSS treated mice (Figure 1E). In addition, plasma G-CSF, IL-6, and IL-17 were significantly increased in both female and male mice with acute colitis, while IFN-γ was only significantly increased in males (Figure 1F).

**FIGURE 2:**
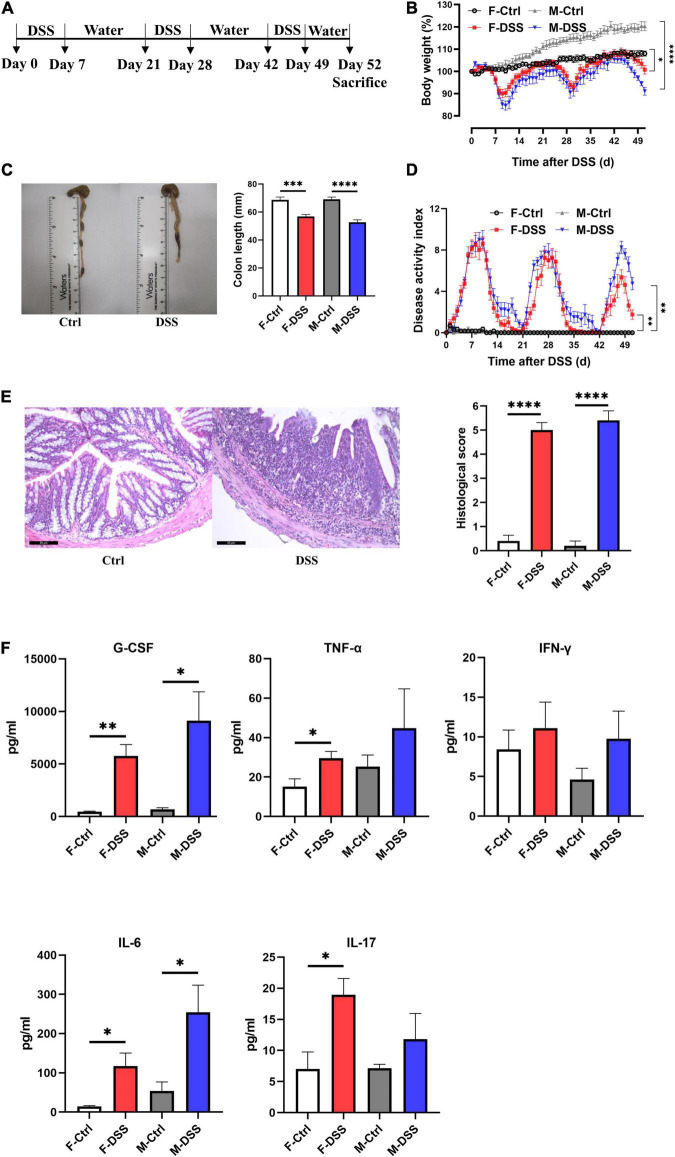
Both female and male mice with chronic DSS-induced colitis displayed significant inflammation in the colon and systemic inflammation. **(A)** Experimental scheme illustrating the experimental protocol with treatment of DSS and water for chronic models. Changes in **(B)** body weight and **(D)** disease activity index (DAI) during DSS treatment. **(C)** Colon length and representative photographs for colon tissue in DSS-treated and control mice. **(E)** Representative images of H&E staining of colon tissue (×200; scale bar, 50 μm), and summary of histological score. **(F)** Levels of plasma cytokines in male and female mice with chronic colitis model. Results are expressed as mean ± SEM. *p < 0.05, **p < 0.01, ***p < 0.001, ****p < 0.0001, in unpaired 2-tailed Student’s *t*-test **(B–F)**, *n* = 5–8 mice each group. F and M, female and male mice, respectively. G-CSF, granulocyte colony-stimulating factor; TNF-α, tumor necrosis factor alpha; IFN-γ, interferon gamma; IL, interleukin.

Chronically DSS-treated mice (3 cycles of 2.5% DSS for 7 days and interspaced with 14 days of drinking water) developed chronic colitis (Figure 2A), as demonstrated by significant decreases in colon length with fluctuating body weight (Figure 2B), enlarged mesenteric lymph nodes (Figure 2C), and fluctuating DAI scoring (Figure 2D), compared to control mice. Increased colonic inflammation was confirmed by histological analysis that showed marked tissue damage and lamina propria inflammatory cell infiltration in colon tissues of mice with chronic colitis, compared to the controls without DSS treatment (Figure 2E). For mice with chronic colitis, plasma G-CSF and IL-6 were significantly increased in both females and males, while TNF-α and IL-17 were increased only in females (Figure 2F).

### Abdominal Aortic Peak Blood Flow Velocity, MILD, PWV, Distensibility, and Radial Strain Were Preserved in Both Male and Female Mice With Acute and Chronic Colitis

**FIGURE 3:**
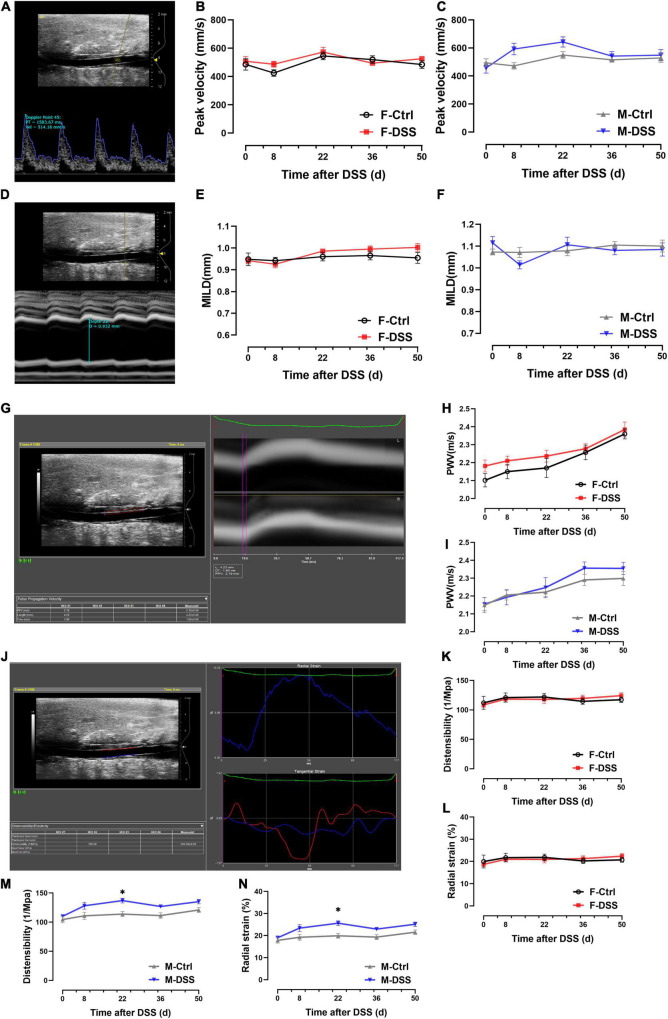
No significant changes in hemodynamics and arterial stiffness of abdominal aorta were observed in both male and female mice with DSS-induced acute and chronic colitis. **(A–C)** Analysis of PW doppler mode images for peak velocity. **(D–F)** Analysis of M-mode images for MILD. **(G–I)** Analysis of EKV images for PWV. **(J–N)** Analysis of EKV images for distensibility and radial strain. Results are expressed as mean ± SEM. *p < 0.05, in two-way ANOVA followed by Bonferroni correction **(E–N)**, *n* = 6–8 mice each group. F and M, female and male mice, respectively. MILD, maximal intraluminal diameter; PWV, pulse wave velocity.

To determine if acute and chronic DSS-induced colitis could affect hemodynamics and arterial stiffness *in vivo*, transabdominal ultrasound imaging of abdominal aorta was conducted at baseline and day 8, 22, 36, and 50 after DSS administration (Supplementary Figure 1). Peak velocity was measured locally in the abdominal aorta using PW doppler mode images and MILD was measured using M-mode images, while PWV, distensibility and radial strain were measured by analyzing EKV data. There were no differences in peak velocity, MILD and PWV of abdominal aorta in either male and female mice with and without DSS-induced colitis (Figures 3A–I). Except in male mice at day 22 after DSS treatment, no differences in distensibility and radial strain were observed in abdominal aorta from either male or female mice with or without DSS-induced colitis (Figures 3J–N). There were no differences in heart rate, respiratory rate and blood pressure in either male and female mice with and without DSS-induced colitis (Supplementary Figure 2).

### Aortic Endothelium-Dependent Vasodilation Was Preserved in Both Male and Female Mice With Acute Colitis

**FIGURE 4:**
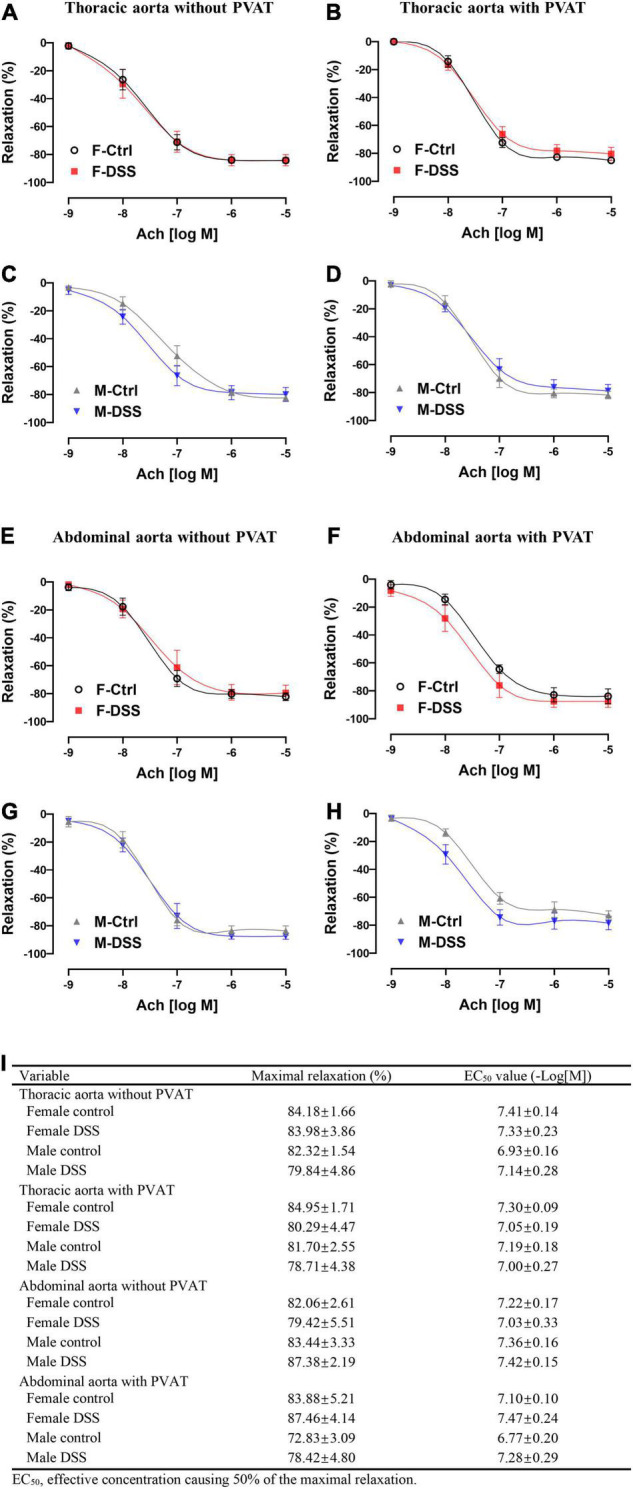
Aortic endothelial function was preserved in both male and female mice with DSS-induced acute colitis. **(A–D)** Ach-induced endothelium-dependent relaxation of thoracic aorta with or without PVAT. **(E–H)** Ach-induced endothelium-dependent relaxation of abdominal aorta with or without PVAT. **(I)** Maximal relaxation and EC_50_ for Ach were calculated in the table. Results are expressed as means ± SEM. *p < 0.05, in two-way ANOVA followed by Bonferroni correction **(A–H)**, *n* = 5–8 mice in each group. PVAT, perivascular adipose tissue; F and M, female and male mice, respectively. Ach, acetylcholine.

To determine if acute DSS-induced colitis could negatively affect aortic endothelial function in mice, endothelium-intact rings of both thoracic and abdominal aorta from both male and female mice were evaluated for endothelium-dependent and endothelium-independent relaxation after submaximal contraction with PE. There were no differences in Ach-induced endothelium-dependent relaxation (Figure 4) or NTG-induced endothelium-independent relaxation (Supplementary Figure 5) of thoracic and abdominal aorta in either male and female mice with and without acute DSS-induced colitis. No differences in PE-induced contraction were observed in either thoracic or abdominal aorta from either male or female mice with or without PVAT in acute DSS-induced colitis (Supplementary Figure 3).

### Endothelium-Dependent Vasodilation Was Selectively Impaired in Abdominal Aorta in Female Mice With Chronic Colitis

**FIGURE 5:**
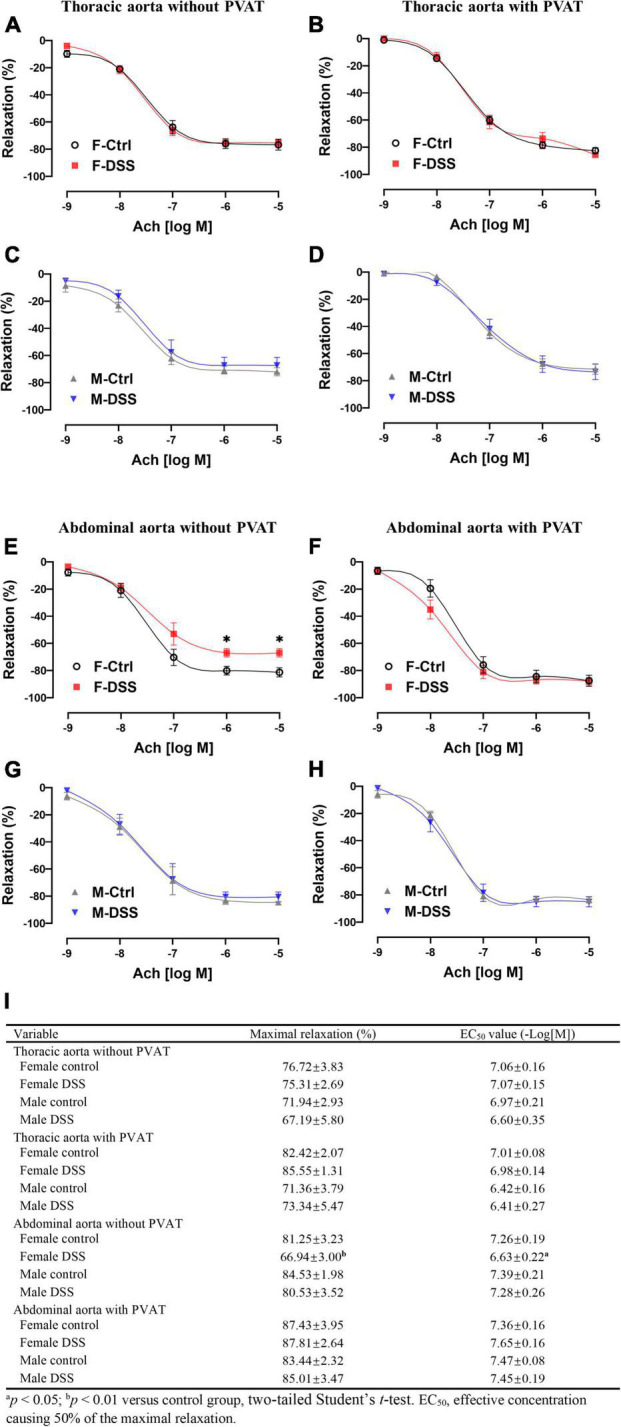
Ach-induced endothelium-dependent relaxation of abdominal aorta was selectively impaired in female mice with DSS-induced chronic colitis. **(A–D)** Ach-induced endothelium-dependent relaxation of thoracic aorta with or without PVAT. **(E–H)** Ach-induced endothelium-dependent relaxation of abdominal aorta with or without PVAT. **(I)** Maximal relaxation and EC_50_ for Ach were calculated in the table. Results are expressed as means ± SEM. *p < 0.05, in two-way ANOVA followed by Bonferroni correction **(A–H)**, *n* = 5–8 mice in each group. PVAT, perivascular adipose tissue; F and M, female and male mice, respectively. Ach, acetylcholine.

To assess aortic endothelial function in chronic colitis, endothelium-intact rings of thoracic and abdominal aorta were precontracted sub-maximally with PE as above. Ach-induced endothelium-dependent relaxation (Figures 5A–D) and NTG-induced endothelium-independent relaxation(Supplementary Figure 6A–D) of thoracic aorta remained intact in both male and female mice with DSS-induced chronic colitis. However, Ach-induced endothelium-dependent relaxation of abdominal aorta was significantly reduced in female mice but not in male mice with chronic colitis mice (Figures 5E,G). The maximal relexation in the control mice was 81.25 ± 3.23% that was significantly reduced to 66.94 ± 3.00% in chronic female colitis mice (*p* = 0.006, *n* = 8 mice per group). The EC_50_ values (-Log[M]) for Ach-induced relaxation of abdominal aorta was significantly increased in female mice with chronic colitis over the control (6.63 ± 0.22 versus 7.26 ± 0.19; *p* = 0.047) (Figure 5I). In contrast, no differences were observed in NTG-induced endothelium-independent relaxation (Supplementary Figure 6E–H). No difference in PE-induced contraction was observed in either thoracic or abdominal aorta from either male or female mice with or without PVAT in chronic DSS-induced colitis (Supplementary Figure 4).

### Perivascular Adipose Tissue Restored Endothelium-Dependent Vasodilation of Abdominal Aorta in Female Mice With Chronic Colitis

To determine if PVAT could have a significant impact on endothelial function in mice with chronic colitis, endothelium-intact rings of thoracic and abdominal aorta with or without PVAT were sub-maximally precontracted with PE. Ach-induced endothelium-dependent relaxation of abdominal aorta without PVAT was significantly reduced in female mice with chronic colitis (Figure 5E) without change in NTG-induced endothelium-independent relaxation (Supplementary Figure 6E). Interestingly, Ach-induced endothelium-dependent relaxation of abdominal aorta was preserved when PVAT was present (Figure 5F).

### ROS Levels Remained Unchanged in Thoracic and Abdominal Aorta in Mice With Either Acute or Chronic Colitis

**FIGURE 6:**
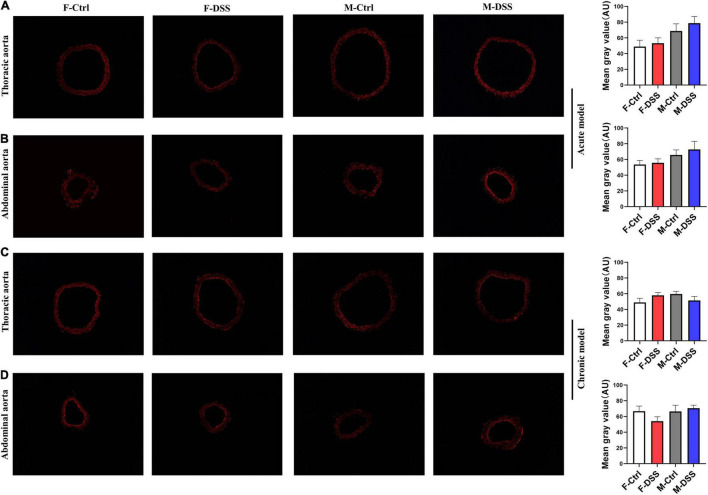
Reactive oxygen species (ROS) levels remained unchanged in thoracic and abdominal aorta in mice with acute and chronic colitis in both female and male mice. Representative images of ROS in acute colitis model **(A,B)** and chronic colitis model **(C,D)** for thoracic and abdominal aortic rings using DHE staining (×100) and graph for quantification of ROS levels in the aortic rings. Results are expressed as means ± SEM. *p < 0.05, in unpaired 2-tailed Student’s *t*-test **(A–D)**, *n* = 5 mice in each group. DHE, dihydroethidium. AU, arbitrary units. F and M, female and male mice, respectively.

Both acute and chronic colitis trigger a systemic inflammation with increased ROS production. Thus, frozen aortic cross-sections were prepared for ROS measurement using DHE. To our surprise, there were no significant differences in ROS levels in fresh aortic cross-sections of either thoracic and abdominal aorta in male and female mice with acute or chronic colitis (Figures 6A–D), while ROS levels were significantly increased in the livers of both male and female mice with DSS-induced chronic colitis (Supplementary Figure 7).

## Discussion

In the present study, we demonstrated that: (1) DSS treatment successfully induced acute and chronic colitis in mice with significant increases in plasma pro-inflammatory cytokines; (2) no significant changes in arterial stiffness was observed in either male and female mice with acute and chronic DSS-induced colitis; (3) no significant changes in endothelium-dependent and endothelium-independent relaxation of both thoracic and abdominal aorta were observed in either male and female mice with acute DSS-induced colitis; (4). endothelium-dependent relaxation was selectively impaired in the abdominal aorta of female mice with DSS-induced chronic colitis but not in males; (5) PVAT preserved endothelium-dependent relaxation of abdominal aorta in female mice with chronic colitis; and (6) no changes in ROS levels were observed in either thoracic and abdominal aorta from male or female mice with acute or chronic colitis. These data suggest that endothelium-dependent relaxation is selectively attenuated in abdominal aorta of female mice with DSS-induced chronic colitis via a PVAT-associated mechanism that is independent of ROS formation.

Systemic inflammation and endothelial dysfunction are considered among the key factors that are critically involved in the development and progression of CVDs. IBD is characterized by chronic and recurrent inflammation of the gastrointestinal tract, and is associated with significant EIMs including non-infectious systemic inflammation and increased risk for CVD, especially in women (3, 4, 22). The data from the present study indeed showed plasma G-CSF, IL-6, and IL-17 levels to be significantly increased in both female and male mice with acute colitis, while IFN-γ was only significantly increased in males. For mice with chronic colitis, plasma G-CSF and IL-6 were significantly increased in both females and males, while TNF-α and IL-17 levels were selectively increased in female mice. These data confirmed that the plasma levels of pro-inflammatory cytokines were increased in mice with acute or chronic colitis.

Intact endothelial function is critical to normal vascular function. Studies have shown that the intestinal microvasculature of IBD patients with inflammatory flare exhibits significant endothelial dysfunction as evident by loss of Ach-induced endothelium-dependent relaxation (23). Surprisingly, there are very limited data on defining the relationship between IBD and endothelial dysfunction in animal models. It has been shown that mice and rats with UC have a significant endothelial damage with increased vascular permeability and perivascular edema in colonic mucosa (24). Mice with DSS-induced colitis and T-cell transfer are reported to exhibit a significant decrease in retinal blood flow (25, 26). The data from the present study showed that *ex vivo* abdominal aortic endothelial function was significantly attenuated in female mice with DSS-induced chronic colitis, while the thoracic aortic endothelial function was preserved. The mechanism(s) for this finding is unclear at this point. It is known that thoracic and abdominal aorta are heterogeneous with different origins during embryogenesis (27). A recent study using different aortic segments and single-cell RNA-sequencing has shown that the cell populations in mouse aorta to be very diverse/heterogeneous. The composition of aortic cell populations, their gene expression profiles and intercellular signaling networks could dramatically and differentially change segmental aortic responses to changes in local and systematic conditions including hemodynamic and metabolic factors (including fats, ions, and glucose) (15). Further studies are needed to define the mechanisms on the selective effect of DSS-induced chronic colitis on abdominal aorta and the apparent sex differences.

One of the interesting findings in the present study is that the endothelial function of abdominal aorta in female mice with DSS-induced chronic colitis is preserved *in vivo*, as is the *ex vivo* function of aortic rings with PVAT. In rodents, mesenteric arteries are surrounded with white adipose tissue, while the thoracic aorta is surrounded with brown adipose-like tissue and the PVAT of abdominal aorta is a mixture of white and brown adipose tissues (28). Historically, PVAT was usually removed from the vascular preparations for *ex vivo* vascular function studies. PVAT is juxtaposed to the vascular adventitia and is now considered to be important for vascular function. PVAT is comprised of dynamic cell populations including nerve terminals and immune cells in addition to adipocytes (29). These cells could directly communicate and interact with vascular cells including adventitial fibroblasts, vascular smooth muscle cells, and endothelial cells (28), thus participating in the regulation of vascular function. Clinical studies have demonstrated that perivascular nerve fiber subclasses are changed in colonic, mesenteric and submucosal blood vessels of IBD patients (30). Animal studies have shown that perivascular sensory neurotransmitter function of mesenteric arteries is profoundly impaired in an IL-10 knock out mouse colitis model (31). The findings in the present study suggests that abdominal aortic PVAT plays an important protective role in relation to endothelial function in female mice with DSS-induced chronic colitis. Further studies are needed to investigate the molecular mechanisms of PVAT in vascular endothelial function in different anatomical locations in IBD and sex difference.

There are significant sex differences in incidence and prevalence, clinical course, EIMs and response to therapies in IBD. Epidemiological studies have shown a greater predominance and severity of CD in women than in men, but a greater predominance and severity of UC in men than in women (32, 33). EIMs are diverse and commonly involve skin, joints, eye, liver and the cardiovascular system. Female IBD patients carry a higher risk of CVDs and anemia, while male patients have a higher risk of primary sclerosing cholangitis and primary ankylosing spondylitis (34–36). Several population studies have shown that women with IBD are at higher risk for acute arterial thrombotic events compared with men with IBD (3, 4). The mechanisms for sex differences in EIMs are unclear. Sex hormones not only affect gut-specific function, including gastric contractility, gastrointestinal transit, and sensitivity to pain (37), but are also important for endothelial function. Estrogen contributes significantly to the regulation of vasomotor activities through estrogen receptors α and β on endothelial cells and vascular smooth muscle cells (38). Physiological levels of testosterone are beneficial to cardiovascular system, while decreased serum levels of testosterone are associated with impaired endothelial function (39). The present study showed that endothelial function is impaired selectively in female mice with chronic colitis. As sexual dimorphism has been implicated in a number of vascular diseases, further studies, using female mice with oophorectomy or male mice with estrogen treatment, are needed to determine if sex hormones contribute to the sex difference in endothelial function observed in mice with chronic colitis.

Significant production of a variety of pro-inflammatory cytokines occurs in IBD, both locally and systemically. In the present study, we observed that plasma TNF-α and IL-17 were selectively increased in female mice with chronic colitis. Increased levels of TNF-α could increase ROS production, and lead to endothelial dysfunction (11, 40). IL-17 is also a potent pro-inflammatory cytokine and activates RhoA/Rho-kinase, leading to endothelial dysfunction (41). However, the ROS levels in both thoracic and abdominal aorta were not increased in either male mice or females with acute and chronic DSS-induced colitis, suggesting that abdominal aortic endothelial dysfunction in female mice with DSS-induced chronic colitis may not be related to ROS formation. Further studies are warranted to explore the mechanism(s) by which abdominal aortic endothelial dysfunction occurs in female mice with chronic colitis.

In conclusion, the present study demonstrated that abdominal aortic endothelial function was attenuated selectively in female mice with DSS-induced chronic colitis independent of ROS formation, and PVAT played an important role in preserving endothelial function in female mice with chronic colitis.

## Data Availability Statement

The raw data supporting the conclusions of this article will be made available by the authors, without undue reservation.

## Ethics Statement

The animal study was reviewed and approved by Institutional Animal Care and Use Committee of the University of Missouri.

## Author Contributions

HW, TH, and ZL contributed to conception and design of the study. HW, TH, MW, ZS, HH, and YC carried out the experiments. LZ and XX performed the statistical analysis. HW wrote the first draft of the manuscript. TH, XL, QZ, ZS, ARP, D-PL, MAH, and CX wrote sections of the manuscript. ZL supervised the project. All authors contributed to manuscript revision, read, and approved the submitted version.

## Conflict of Interest

The authors declare that the research was conducted in the absence of any commercial or financial relationships that could be construed as a potential conflict of interest.

## Publisher’s Note

All claims expressed in this article are solely those of the authors and do not necessarily represent those of their affiliated organizations, or those of the publisher, the editors and the reviewers. Any product that may be evaluated in this article, or claim that may be made by its manufacturer, is not guaranteed or endorsed by the publisher.
