# Supplementary material for: Abdominal Aortic Endothelial Dysfunction Occurs in Female Mice With Dextran Sodium Sulfate-Induced Chronic Colitis Independently of Reactive Oxygen Species Formation
Source: Front Cardiovasc Med. 2022 Apr 7;9:871335. doi: 10.3389/fcvm.2022.871335 (PMC9021429; doi:10.3389/fcvm.2022.871335)
Supplement: Supplementary file 1 [file Data_Sheet_1.docx]

**Supplementary Materials:**


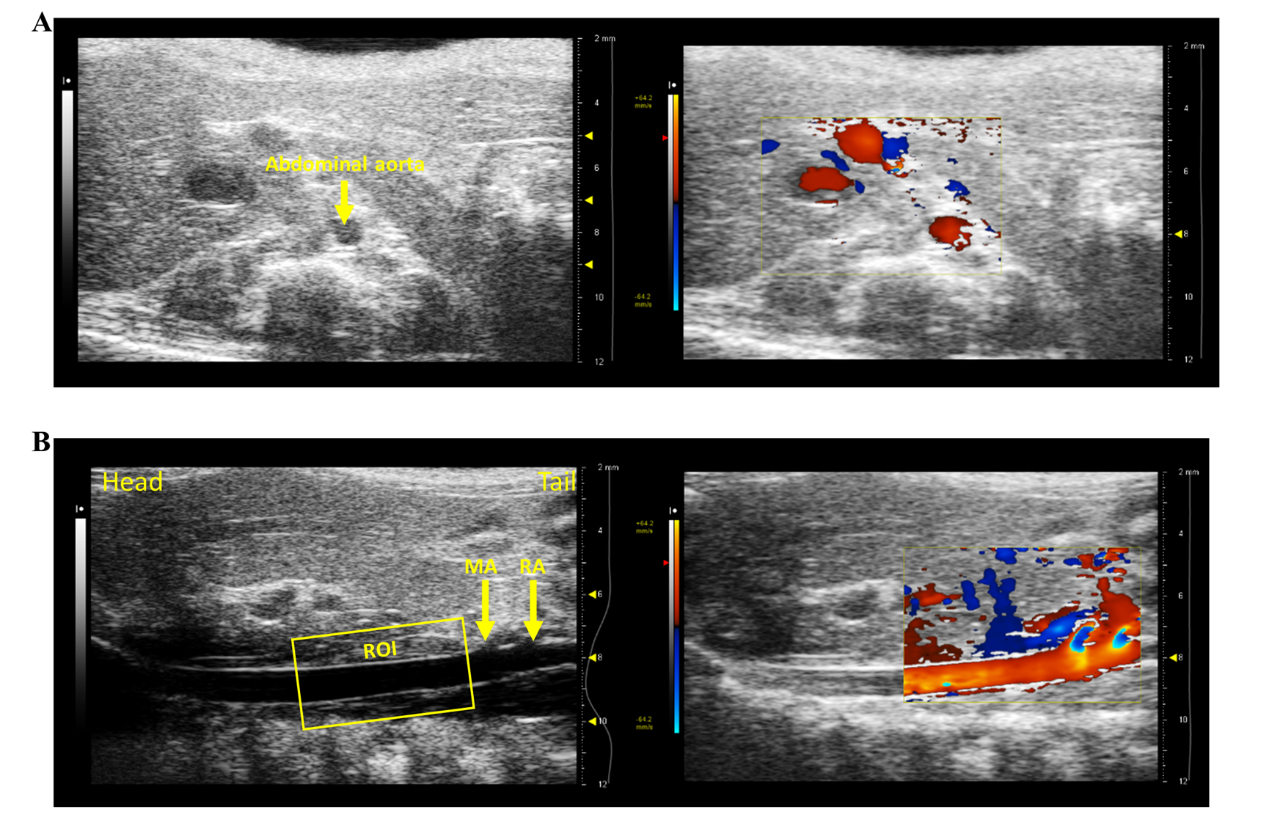


**Figure S1.** **Ultrasound imaging of the abdominal aorta.** (**A**) Transverse view of abdominal aorta. (**B**) Longitudinal view of abdominal aorta. ROI, region of interest; MA, mesenteric artery; RA, renal artery.


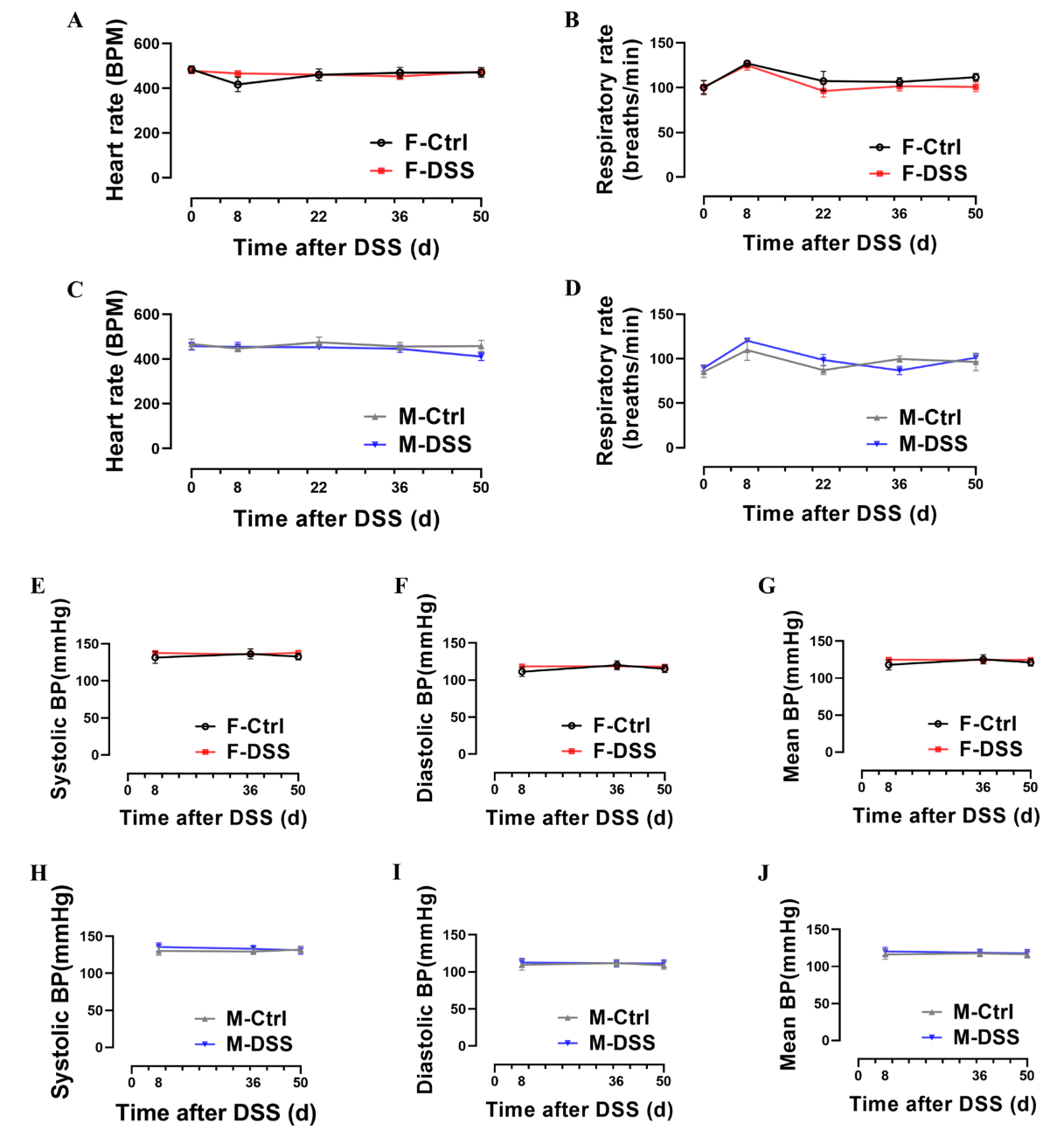


**Figure S2. No changes in heart rate, respiratory rate and blood pressure were observed in both male and female with DSS-induced acute or chronic colitis.** (**A-D**) Heart rate and respiratory rate for mice with or without DSS-induced acute or chronic colitis. (**E-J**) Systolic, diastolic and mean BP for the mice without general anesthesia. Results were expressed as mean ± SEM. **p* < 0.05, with two-way ANOVA followed by Bonferroni correction (**A-J**), n=6-8 mice each group. **F and M**, female and male mice, respectively. **BPM**, beats per minute; **BP**, blood pressure; **EKV**, ECG-based Kilohertz Visualization.


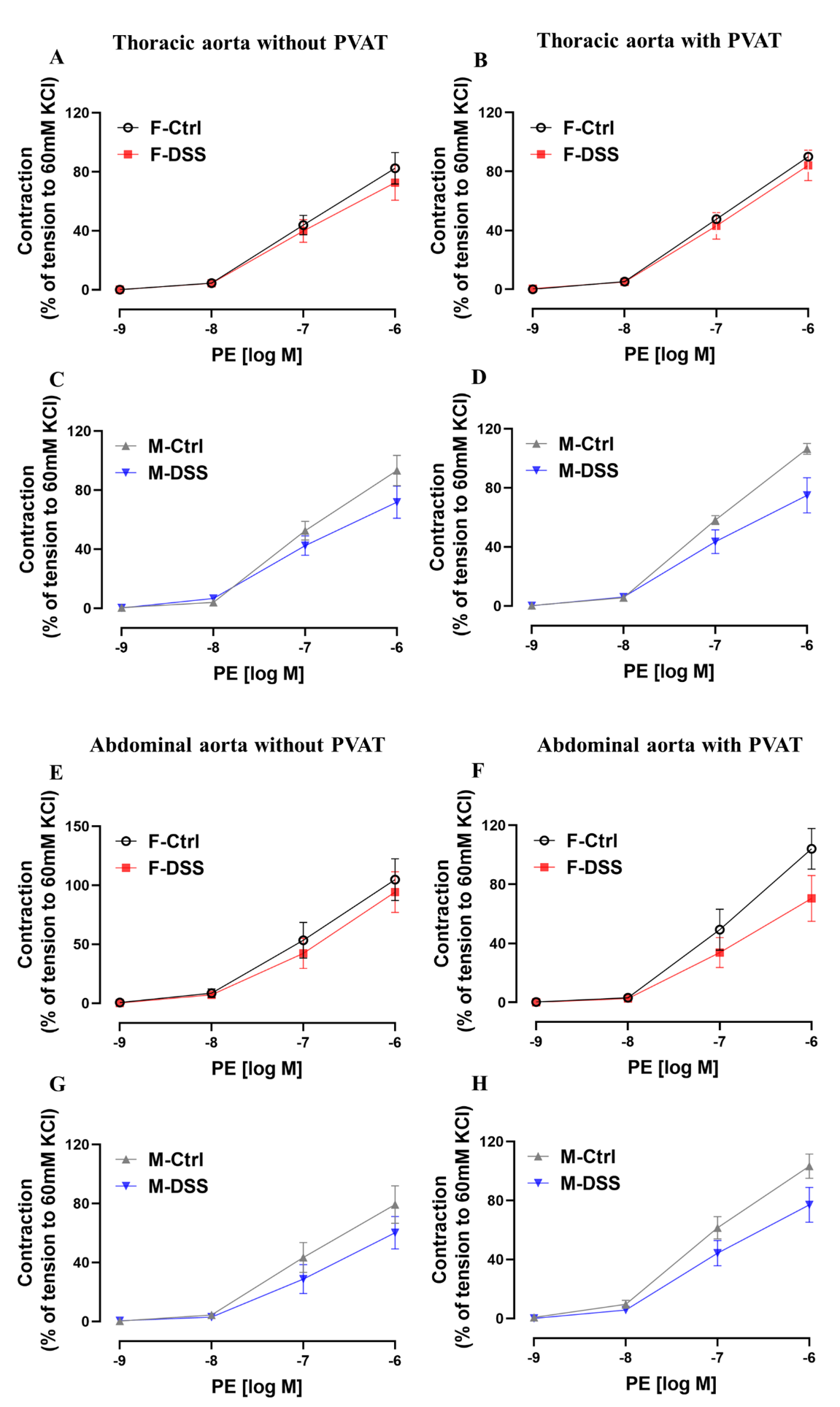


**Figure S3. No difference in aortic contractility to cumulative dosing of PE was observed in both male and female with DSS-induced acute colitis.** (**A-D**) PE-induced contractility of thoracic aorta with or without PVAT. (**E-H**) PE-induced contractility of abdominal aorta with or without PVAT. Results are expressed as means ± SEM. **p*< 0.05, in two-way ANOVA followed by Bonferroni correction (A-H), n=5-8 mice in each group. PVAT, perivascular adipose tissue; F and M, female and male mice, respectively. PE, phenylephrine.


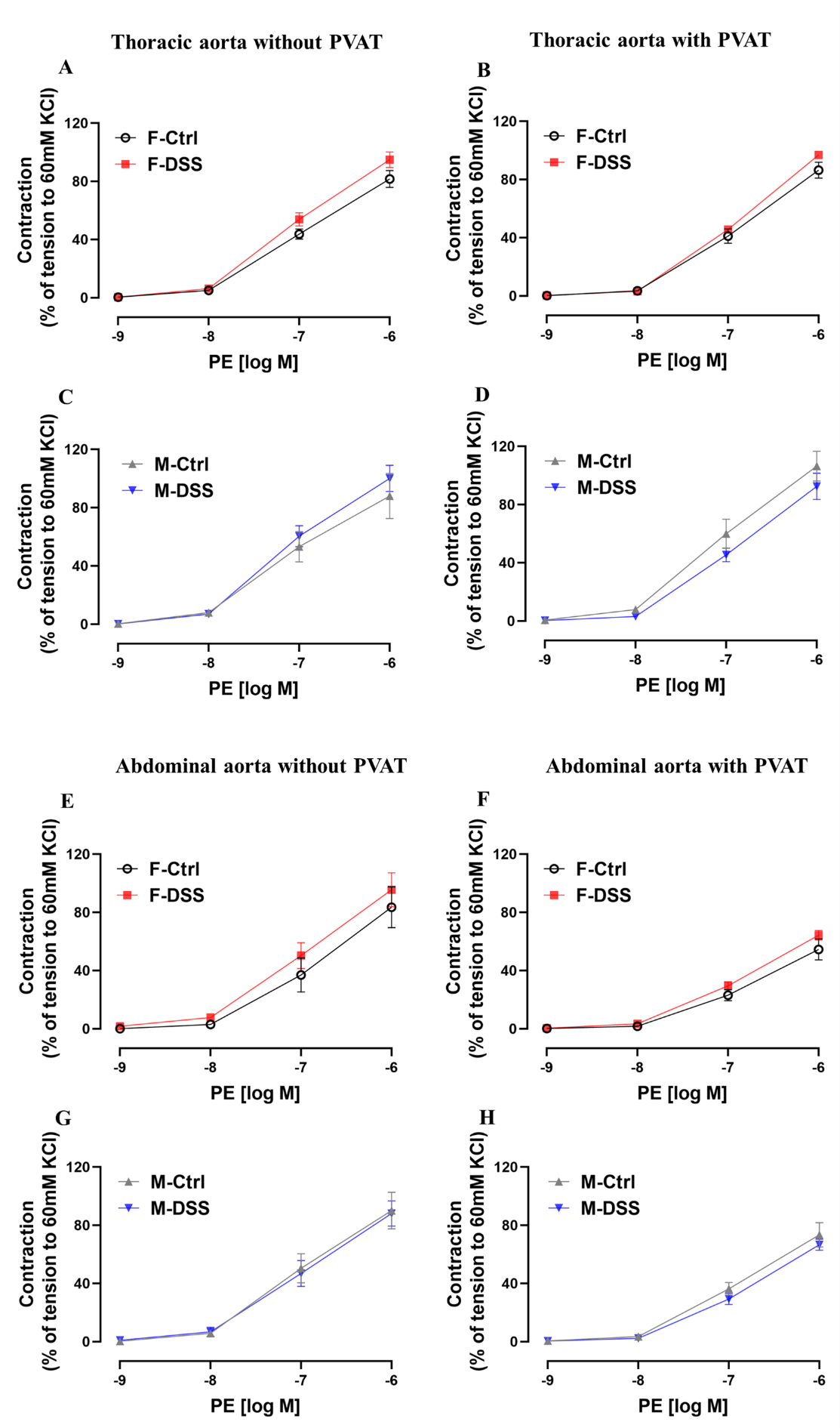


**Figure S4. No change in aortic contractility to cumulative dosing of PE in both male and female mice with DSS-induced chronic colitis.** (**A-D**) PE-induced contractility of thoracic aorta with or without PVAT. (**E-H**) PE-induced contractility of abdominal aorta with or without PVAT. Results are expressed as means ± SEM. **p* < 0.05, in two-way ANOVA followed by Bonferroni correction (A-H), n=5-8 mice in each group. PVAT, perivascular adipose tissue; F and M, female and male mice, respectively. PE, phenylephrine.


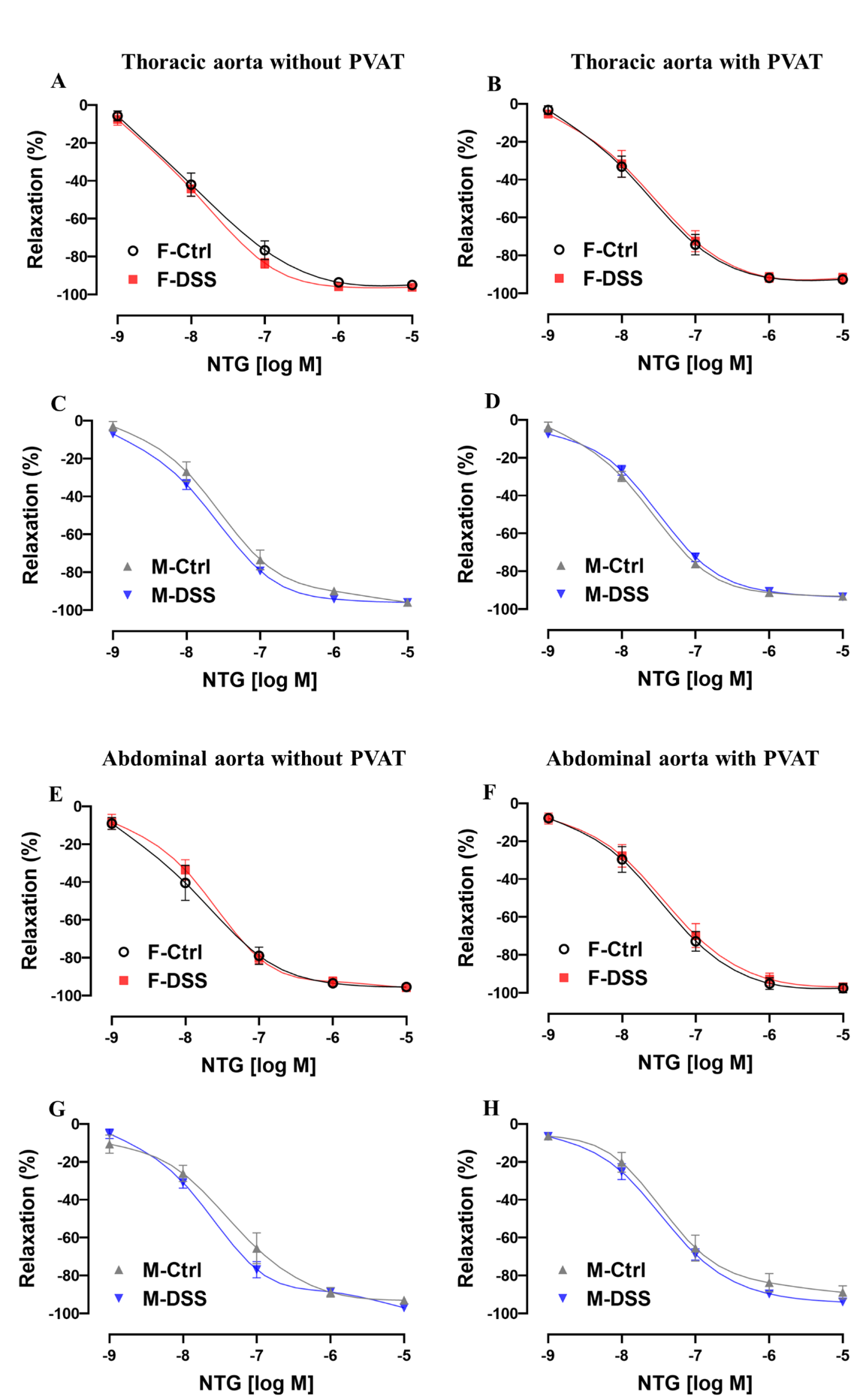


**Figure S5. There was no change in NTG-induced endothelium-independent relaxation of thoracic and abdominal aorta in both male and female mice with DSS-induced acute colitis.** (**A-D**) NTG-induced endothelium-independent relaxation of thoracic aorta with or without PVAT. (**E-H**) NTG-induced endothelium-independent relaxation of abdominal aorta with or without PVAT. Results are expressed as means ± SEM. **p* < 0.05, in two-way ANOVA followed by Bonferroni correction (A-H), n=5-8 mice in each group. PVAT, perivascular adipose tissue; F and M, female and male mice, respectively. NTG, nitroglycerin.


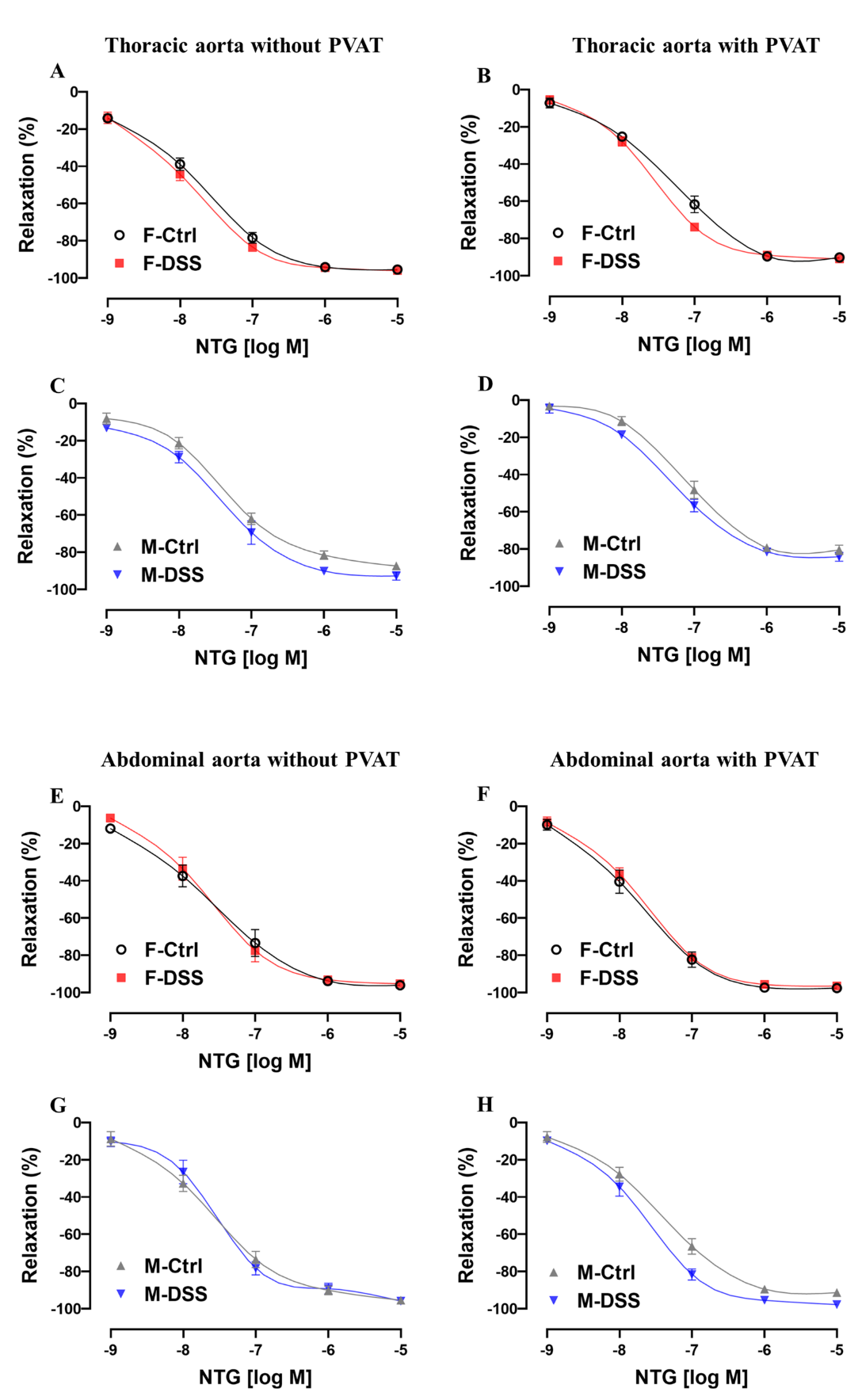


**Figure S6. No change in NTG-induced endothelium-independent relaxation of thoracic and abdominal aorta was observed in both male and female mice with DSS-induced chronic colitis.** (**A-D**) NTG-induced endothelium-independent relaxation of thoracic aorta with or without PVAT. (**E-H**) NTG-induced endothelium-independent relaxation of abdominal aorta with or without PVAT. Results are expressed as means ± SEM. **p* < 0.05, in two-way ANOVA followed by Bonferroni correction (A-H), n=5-8 mice in each group. PVAT, perivascular adipose tissue; F and M, female and male mice, respectively. NTG, nitroglycerin.


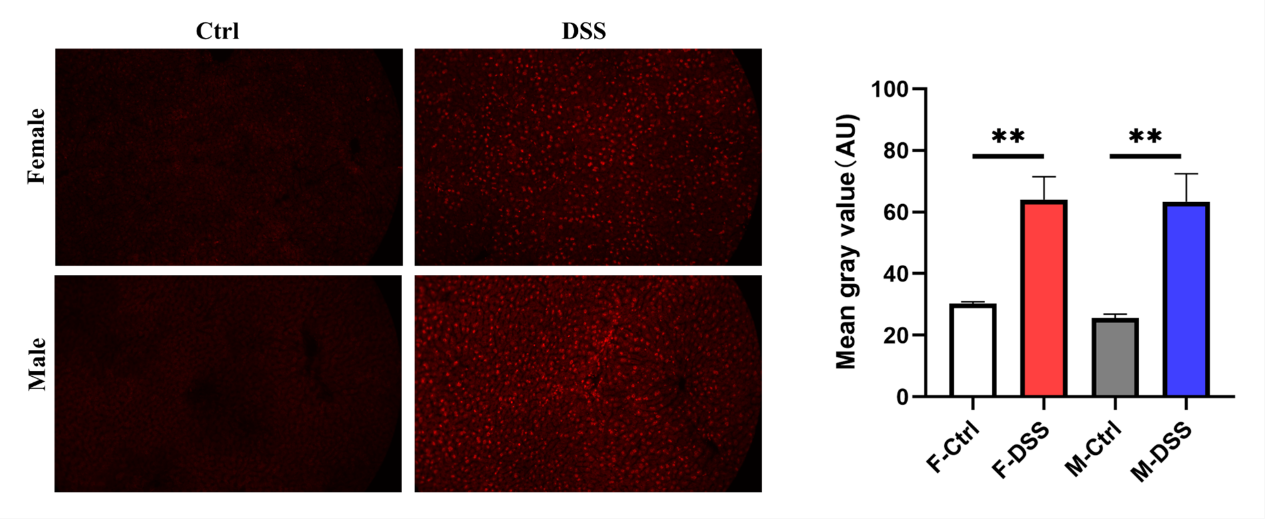


**Figure S7. ROS level was significantly increased in the liver of both female and male mice with chronic colitis**. Representative images of ROS in the liver of mice with chronic colitis using DHE staining (×100) and graph for quantification of ROS levels. Results are expressed as means ± SEM. ***p* < 0.01, in unpaired 2-tailed Student’s *t* test, n=5 mice in each group. DHE, dihydroethidium. AU, arbitrary units.
